# The New Frontier of Functional Genomics: From Chromatin Architecture and Noncoding RNAs to Therapeutic Targets

**DOI:** 10.1177/2472555220926158

**Published:** 2020-06-02

**Authors:** Natali Papanicolaou, Alessandro Bonetti

**Affiliations:** 1 Division of Biomaterials, Department of Medical Biochemistry and Biophysics, Karolinska Institutet, Stockholm, Sweden; 2 Laboratory of Molecular Neurobiology, Department of Medical Biochemistry and Biophysics, Karolinska Institutet, Stockholm, Sweden; 3 RIKEN Center for Integrative Medical Sciences, Yokohama, Kanagawa, Japan

**Keywords:** lncRNA, enhancer, GWAS, chromatin

## Abstract

Common diseases are complex, multifactorial disorders whose pathogenesis is influenced by the interplay of genetic predisposition and environmental factors. Genome-wide association studies have interrogated genetic polymorphisms across genomes of individuals to test associations between genotype and susceptibility to specific disorders, providing insights into the genetic architecture of several complex disorders. However, genetic variants associated with the susceptibility to common diseases are often located in noncoding regions of the genome, such as tissue-specific enhancers or long noncoding RNAs, suggesting that regulatory elements might play a relevant role in human diseases.

Enhancers are *cis*-regulatory genomic sequences that act in concert with promoters to regulate gene expression in a precise spatiotemporal manner. They can be located at a considerable distance from their cognate target promoters, increasing the difficulty of their identification. Genomes are organized in domains of chromatin folding, namely topologically associating domains (TADs). Identification of enhancer–promoter interactions within TADs has revealed principles of cell-type specificity across several organisms and tissues.

The vast majority of mammalian genomes are pervasively transcribed, accounting for a previously unappreciated complexity of the noncoding RNA fraction. Particularly, long noncoding RNAs have emerged as key players for the establishment of chromatin architecture and regulation of gene expression.

In this perspective, we describe the new advances in the fields of transcriptomics and genome organization, focusing on the role of noncoding genomic variants in the predisposition of common diseases. Finally, we propose a new framework for the identification of the next generation of pharmacological targets for common human diseases.

## Introduction

Common human diseases such as cancer, cardiovascular disorders, diabetes, and mental illness represent the major contributors to morbidity and mortality in both the developed and developing world. Although our understanding for the pathogenic mechanisms underlying most common diseases has significantly improved over the last decades, the prevention and treatment of these disorders are still largely ineffective.

Most common diseases are highly heterogeneous, with few cases characterized by simple etiology dominated by a single genetic mutation, while the most cases are caused by the interplay of multiple genetic and environmental factors. Before the completion of the Human Genome Project, efforts to map genetic variants were mostly based on linkage studies that searched for shared haplotypes among related affected individuals. However, linkage studies do not have enough power to detect common variants with modest effect, and they have been slowly replaced by association studies.^1^ Initially scientists focused on association studies between variation in candidate genes and common diseases. With the development of international efforts such as the HapMap project, the scientific community rapidly moved from probing variation present in a few candidate genes to testing millions of single-nucleotide polymorphisms (SNPs) located throughout the genome.^2^

Genome-wide association studies (GWAS) provide a genetic approach to identifying molecular pathways involved in complex traits and diseases by defining associations between genetic variants and phenotypes of interest.^3,4^ Large-scale GWAS have discovered hundreds of SNPs that are associated with the risk of common disorders. However, the majority of disease-risk variants are located outside protein-coding genes, suggesting a role for noncoding variation in the human genome.^4^

The emergence of next-generation sequencing technologies combined with genome-wide approaches has led to the identification of key biological regulators located in noncoding regions of the human genome. Advances in the field of genomics and transcriptomics have uncovered the role of DNA and RNA elements, such as enhancer regions and long noncoding RNAs (lncRNAs), in the fine-tuning of crucial physiological processes. Specifically, these studies have shown that genetic polymorphisms located in such regions have a regulatory role and, hence, a subtler impact on the pathogenesis of human diseases when compared with mutations underlying mendelian disorders. Altogether, these recent findings highlight the need for a new approach to systematically study the functional outcome of noncoding polymorphisms present in the genome.

In this perspective, we describe the latest findings about emerging regulatory roles of noncoding elements in the human genome and their association with the predisposition to common diseases. We mention the latest technological advances that allow researchers to unveil the functional role of genetic variants located in different genomic regions. Finally, we propose a new framework to investigate the functional role of noncoding variation in the genome and, in turn, to identify new pharmacological and therapeutic targets.

## Noncoding Regions of the Human Genome

### Enhancers

Chromosomal organization is an evolutionarily conserved feature that requires hierarchical folding of the chromatin into large compartments composed of smaller domains called topologically associating domains (TADs). TADs are stable across cell divisions, invariant across different cell types, and evolutionarily conserved, indicating their fundamental importance in chromatin organization.^5^ A key feature of most TAD boundaries is the presence of the CCCTC binding factor (CTCF) together with the structural maintenance of the chromosome cohesion complex.^5^ The binding of CTCF at TAD borders is crucial, as removal one of its binding sites is sufficient to abrogate a TAD boundary.^6^ Notably, intra-TAD interactions between regulatory elements are cell type specific and investigation of DNA-DNA contacts with high-throughput sequencing technologies can reveal multiple layers of chromatin organization and regulation.

Regulation of gene expression generally involves two different types of *cis*-acting elements: the promoter, a genomic region defining the initiation of transcription, and more distal regulatory elements called enhancers. While promoters provide the essential sites of transcriptional initiation of RNAs, they are frequently not sufficient to direct appropriate developmental and signal-dependent levels of gene expression.^7,8^ This additional information is provided by enhancers, short regions of DNA that, when bound by transcription factors (TFs), enhance RNA expression from target promoters. Enhancers can reside hundreds of thousands of base pairs away from their target gene and are typically well conserved across genomes, and their function is generally considered to depend on three-dimensional (3D) enhancer–promoter interactions.^9^ Within TADs, DNA folding allows fine-tuning of gene expression by facilitating contacts between regulatory sequences such as promoters and enhancers. Enhancer selection is driven by cell-type-specific combinations of lineage-determining TFs that, in turn, specify the binding of signal-dependent TFs. As a consequence, each cell has a unique enhancer repertoire that underlies its particular pattern of gene expression and enables cell-type-specific responses to intra- and extracellular signals.^10^ Furthermore, active enhancers are characterized by bidirectional transcription, which results in the production of enhancer RNAs (eRNAs) believed to facilitate long-range enhancer–promoter looping. Accordingly, genetic variation affecting enhancer selection and function is considered to be a major determinant of differences in cell-type-specific gene expression between individuals.

### LncRNAs

Recent international scientific efforts have discovered that the vast majority of mammalian genomes are pervasively transcribed, accounting for a previously unappreciated complexity of the noncoding RNA (ncRNA) fraction.^11,12^ In particular, lncRNAs (lncRNAs) have emerged as important regulators of various biological processes.^13^ LncRNAs are characterized by a length longer than 200 nucleotides and the absence of open reading frames. As such, the definition is very general, and under the name lncRNAs we include different transcripts, such as long intergenic noncoding RNAs (lincRNAs), eRNAs, and sense and antisense RNAs.^14^ Like mRNAs, lncRNAs are transcribed by RNA polymerase II and are capped and polyadenylated. However, compared with protein-coding transcripts, lncRNAs have strict spatiotemporal expression patterns, exhibiting tissue- and cell-type specificity.^15^ Also, unlike coding transcripts that are predominantly cytosolic,^16^ approximately 60% of noncoding transcripts are enriched in the nucleus, specifically in the chromatin fraction,^17,18^ suggesting that their action might depend on their interactions with chromatin and chromatin binding factors.^18^ As the cytoplasmic functions of lncRNAs have been already reviewed elsewhere,^19^ we focus on the nuclear functions of lncRNAs in this perspective.

**Figure 1.**
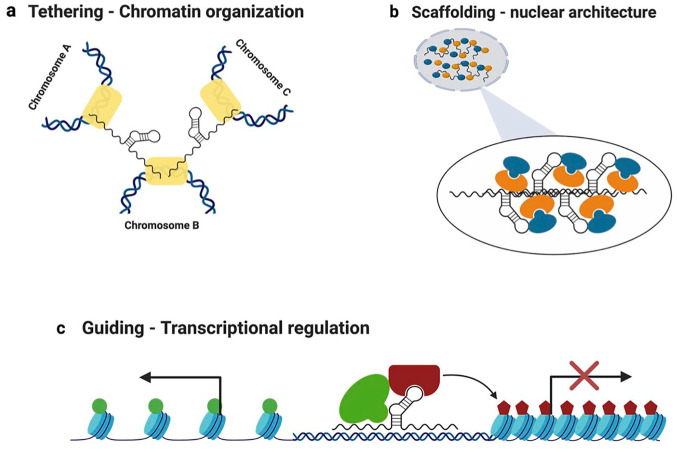
Mechanisms of action of lncRNAs. Schematic representation of the mechanisms of action of lncRNAs. (a) LncRNAs can act as tethers to increase the proximity of spatially distant chromosomes, contributing to chromatin organization. This figure represents how tethering lncRNAs are able to bring three distinct chromosomes into closer proximity, by interacting with their target loci (yellow rectangles) on the respective chromosomes. (b) This figure represents a subnuclear organelle (i.e., nuclear speckle or paraspeckle) formed through interactions between proteins and nucleic acids. Some lncRNAs, such as *MALAT1* and *NEAT1*, function as scaffolds for interacting proteins (orange and blue shapes) and genomic DNA regions, contributing to the formation of the subnuclear organelles. (c) As guides, lncRNAs participate in transcriptional regulation *in cis* or *in trans*, by recruiting chromatin-modifying complexes to their target genes. This figure represents an lncRNA molecule guiding two distinct histone modifier proteins to its target loci. In this example, the histone modifier represented in green mediates transcription-permissive chromatin modifications (shown as green circles on histones). In contrast, the histone modifier represented in red, recruited by the same lncRNA, mediates repressive histone modifications (red pentagons on histones), leading to chromatin condensation and transcriptional inhibition of the target locus.

During recent years, functional classification of lncRNAs has proven to be a challenging task, as we are only now starting to appreciate the multiple mechanisms of action for these transcripts. Nuclear lncRNAs are able to perform diverse functions, such as involvement in nuclear architecture (scaffolding function; **Fig. 1a**), organization of the chromatin structure (tethering function; **Fig. 1b**), and transcriptional regulation (guiding function; **Fig. 1c**), with some lncRNAs, such as *XIST*, being able to perform a combination of functions (scaffolding and guiding). In the next sections, we discuss the different mechanisms of action for nuclear lncRNAs in further detail.

### LncRNAs in Nuclear Architecture

Two of the most well-studied lncRNAs, *NEAT1* (nuclear enriched abundant transcript 1) and *MALAT1* (metastasis-associated lung adenocarcinoma transcript 1; also known as *NEAT2*), have been demonstrated to play important scaffolding roles in the assembly of nuclear paraspeckles and speckles, respectively, by interacting with protein components.^15^ *MALAT1* is a highly abundant nuclear lncRNA that localizes in nuclear speckles by physically interacting with splicing factors such as SRSF1 and SC-35 (aka SRSF2).^20^ Although *MALAT1* has been shown to be dispensable for nuclear speckle formation, it is thought to facilitate the localization of nuclear speckles at transcriptionally active gene loci.^14^

*NEAT1* provides another example of an architectural lncRNA, involved in the formation of nuclear paraspeckles. Paraspeckles are nuclear bodies formed by interacting protein and RNA components, believed to be crucial for mRNA retention, mRNA cleavage, and protein sequestration.^21,22^ In humans, the *NEAT1* transcript is processed to produce two distinct transcripts, a 3.7 kb *NEAT1_1* and a 22.7 kb *NEAT1_2*, often referred to as the short and long *NEAT1* isoforms.^14^ Interestingly, only the longer isoform *NEAT1_2* is essential for paraspeckle assembly,^23^ by interacting with essential paraspeckle protein components such as NONO/P54NRB and SFPQ.^24^

### LncRNAs in Chromatin Organization

The evolutionarily conserved, nuclear, lincRNA *FIRRE* (functional intergenic repeating RNA element) is a representative example of the nuclear organization properties of some lncRNAs. The *FIRRE* locus is localized on the X chromosome and has been observed to escape X chromosome inactivation, localizing to a 5 Mb domain in the vicinity of its site of transcription.^25^ *FIRRE* RNA contains multiple repeats of a 156 nt long sequence that enables binding to the nuclear matrix organizing protein HNRNPU. This lncRNA can establish trans-chromosomal interactions across five distinct loci, bringing in spatial proximity to its site of transcription and contributing to higher-order chromatin organization. Additionally, *FIRRE* RNA has been demonstrated to bind the architectural protein CTCF, targeting the inactive X chromosome in the perinucleolar region.^26^

### LncRNAs in Transcriptional Regulation

Most lncRNAs exhibit nuclear localization with enrichment for the chromatin fraction; furthermore, they have the ability to fold into space and associate with specific proteins such as TFs to fine-tune gene expression.^15^ As such, these RNAs can deliver proteins to specific gene locations and coordinate genetic programs. Multiple studies have shown that lncRNAs can regulate expression of target genes in *cis* (in proximity to their site of transcription or more distantly but on the same chromosome of origin) or in *trans* (on chromosomes different from the chromosome of origin).

Probably the most well-studied example of a *cis*-acting lncRNA is *XIST* (X-inactive specific transcript), a crucial transcript for the process of dosage compensation in female mammal cells. Dosage compensation is an essential process to ensure that gene expression levels from two X chromosomes in female cells are equivalent to gene expression levels from the single X chromosome found in male cells. *XIST* is transcribed from one of the two X chromosomes and physically associates with the Polycomb repressive complex 2 (PRC2) to initiate epigenetic repression of the entire X chromosome through the deposition of H3K27me3 histone marks by EZH2.^27^ Following the epigenetic silencing, the inactive X chromosome localizes to the nuclear periphery.

The HOX antisense intergenic RNA (*HOTAIR*) is a well-known *trans*-acting lncRNA. *HOTAIR* is transcribed in the antisense direction to the *HOXC* locus and located on chromosome 12 in humans. Despite being initially thought to be a *cis*-acting, negative regulator of *HOXC* locus expression, its inhibition does not affect *HOXC* expression. In contrast, *HOTAIR* inactivation leads to a significant upregulation of the *HOXD* locus, located on a different chromosome (chromosome 2 in humans). Similar to *XIST, HOTAIR* interacts with PRC2 to guide the repressive machinery to the HOXD locus and initiate epigenetic silencing through H3K27 trimethylation (H3K27me3).^28^

The versatility of regulatory processes controlled by lncRNAs together with the extensive network of interacting protein partners has revealed the biological importance of these transcripts. Therefore, genetic variation in loci encoding for lncRNAs can significantly affect their functionality and have a role in disease development.

## Genetic Variation in Noncoding Regions Associated with Common Disorders

In this section, we discuss some recently published evidence that highlights the role of genetic polymorphisms located in noncoding regions with the association with common disorders. We have provided a summary table that describes the mechanisms of action for some well-known risk variants (**Table 1**).

**Table 1.** Summary of Noncoding Variants Associated with Common Disorders.

Disorder	Variant In	Affected Genes	Mechanism of Action of Risk Variants	Reference
Systemic lupus erythematosus (SLE)	Enhancer	A20 (*TNFAIP*), *LINC00513*	Disruption of long-range enhancer–promoter interactions	34
Colorectal cancer	Enhancer (rs6983267)	*MYC*	Enhancer activation through differential binding of β-catenin and TCFL2	35
Neuroblastoma	Superenhancer	*LMO1*	Increased binding of the TF GATA3, facilitating long-range enhancer–promoter looping and LMO1 upregulation	36
Prostate cancer	Enhancer (rs11672691)	*PCAT19, CEACAM21*	Stronger binding of HOXA2 leads to upregulation of PCAT19 and CEACAM21	37, 38
Esophageal squamous cell carcinoma Gastric cancer	Enhancer (rs920778)	*HOTAIR*	Variant results in de novo enhancer element upregulating HOTAIR	39, 40
Obesity (adult and childhood) Type 2 diabetes	Enhancer	*FTO, IRX3*	Long-range interactions with obesity-related risk alleles, increasing IRX3 expression	41–43
Adult-onset demyelinating leukodystropy (ADLD)	TAD boundary	*LMNB*	Enhancer adoption by LMNB leading to overexpression	44
Cardiovascular disease (CVD), glaucoma, endometriosis	lncRNA *ANRIL*	*CDKN2A, CDKN2B*	Dysregulation of epigenetic silencing of CDKN2A and CDKN2B	55–58
Myocardial infarction	lncRNA *MIAT*	*MIR-150-5p, MIR-24*	Mechanism of action not well-described; mutations in MIAT gene could lead to aberrant binding of the miR targets	61–63
Celiac disease (CeD)	lncRNA *LNC13* (rs917997)	*IL18RAP*	Inefficient binding of hnRNPD leads to reduced transcriptional repression of IL18RAP and pro-inflammatory gene expression	64

For each disease-associated noncoding variant mentioned in the main text, its genomic location and putative mechanism of action are also reported.

### Variation in Enhancers

**Figure 2.**
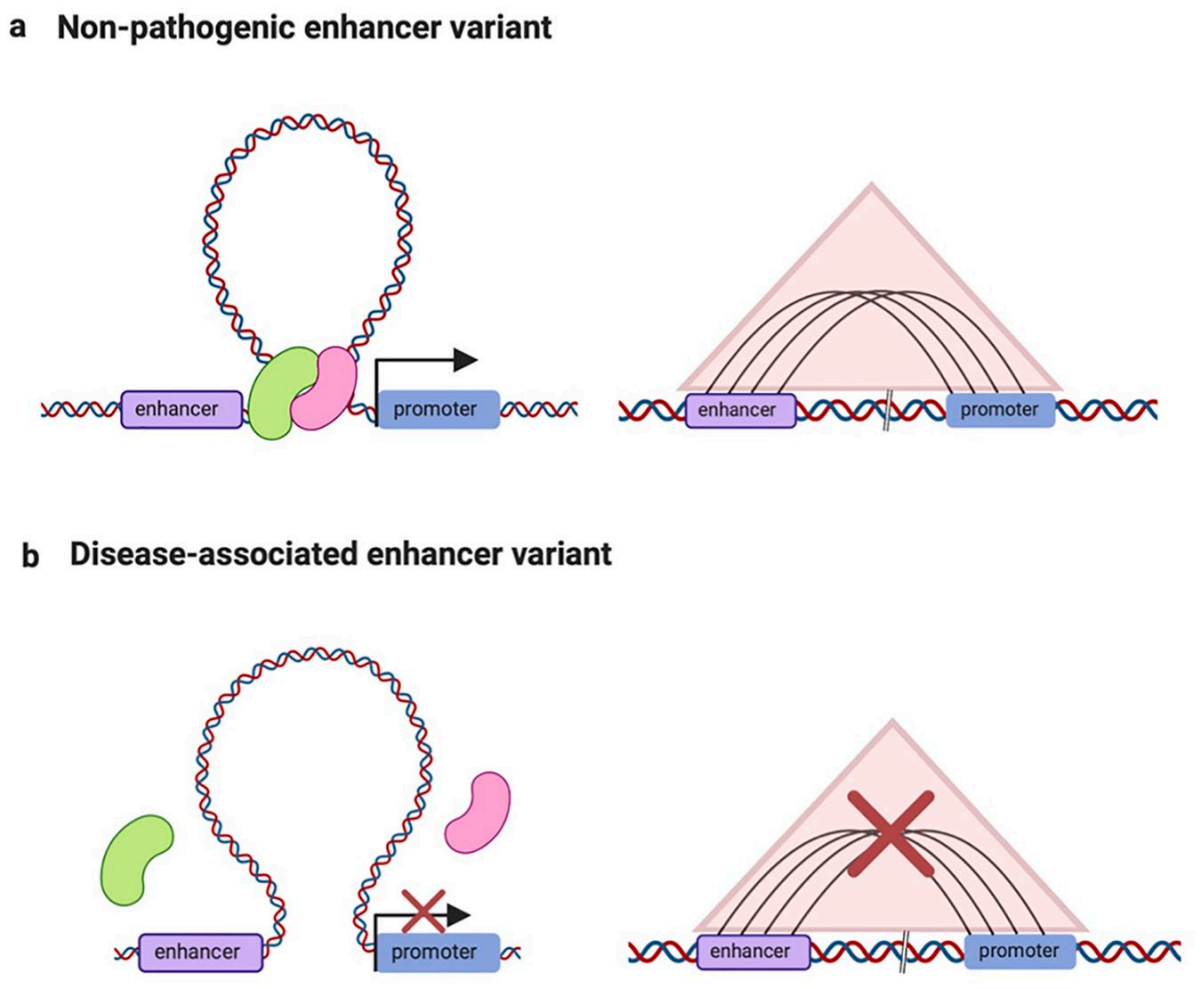
Effects of genetic variations on enhancers. Graphical representation of the effects of genetic variants on the function of enhancers. The contact maps represent the presence or absence of 3D interactions between enhancers and target gene promoters, respectively. (a) The nonpathogenic enhancer variant (purple rectangle) is successfully bound by TFs (pink and green) and brought in proximity to its target gene promoter (blue rectangle) through effective chromatin looping with transcriptional activation of the target. The pyramid in light red represents the hypothetical contact map resulting from effective chromatin looping between the enhancer and the respective target genes. (b) The disease-associated enhancer variant can no longer be bound by the respective TFs (pink and green) or chromatin-modifying complexes, which results in defective chromatin looping and impaired enhancer–promoter interactions, leading to low transcriptional activity of the target genes.

Enhancers represent genomic regulatory elements (GREs) that are crucial for the regulation of distinct spatiotemporal transcriptional programs. Consequently, disease-associated variations within enhancer elements are likely to result in transcriptional dysregulation of cell-type-specific gene expression. Indeed, genetic risk variants are enriched in enhancer-like elements, featuring increased DNAse hypersensitivity sites and the aforementioned histone marks.^29^ **Figure 2** depicts the consequences of genetic variation at enhancer regions in terms of disease predisposition. As presented in **Figure 2a**, the wild-type (WT) enhancer variant is bound by sequence-specific TFs that mediate long-distance enhancer–promoter interactions through chromatin looping. The resulting proximity of enhancer and promoter regions promotes transcriptional activation of the target genes. However, in the case of a disease-associated enhancer variant (**Fig. 2b**), the genetic variant results in impaired TF binding, leading to unsuccessful chromatin looping and lack of enhancer–promoter interactions. In turn, the target gene’s transcriptional activation is inefficient, resulting in basal transcription levels.

Disease-associated enhancer variants are more likely to be found in enhancer sequences that are active in cells and tissues relevant to the disease of interest. For example, pancreatic islet enhancers are specifically enriched in risk variants associated with type 2 diabetes.^30^ In the case of Parkinson’s disease (PD), more than 90% of reported cases present non-Mendelian inheritance. A study by Soldner and colleagues found a major risk allele for PD within an enhancer of the α-synuclein gene, which results in altered TF binding and upregulation of the protein.^31^ Similarly, 30% of noncoding variants associated with Alzheimer’s disease (AD) were found to be located in enhancer elements, with their target genes involved with amyloid-beta plaque clearance, synaptic transmission, and immune responses.^32^ Furthermore, these variants were observed to affect TF and CTCF binding sites, suggesting that their presence might influence AD risk by affecting TF binding and enhancer–promoter chromatin looping mechanisms.^32^ A large class of complex disorders includes autoimmune disorders such as systemic lupus erythematosus (SLE), multiple sclerosis, and type 1 diabetes. In a large study investigating noncoding variants in 21 autoimmune diseases, it was determined that approximately 60% of likely causal variants map to enhancer-like elements that are often stimulus-dependent CD4+ T-cell-specific enhancers.^33^ Additionally, in the case of SLE, two risk variants were found to locate in a distal enhancer, regulating the expression of the NFκB inhibitor A20, encoded by the *TNFAIP* gene. The nonrisk enhancer was found to interact with the promoter of *TNFAIP* through long-range chromatin looping and enhance its expression upon direct binding of NFκB. However, the presence of the risk variants impairs NFκB binding, disrupts the long-range enhancer–promoter interactions, and results in low expression of *TNFAIP*.^34^

Enhancer variants have also been identified in various types of cancers. In the case of colorectal cancer, a variant mapping to the 8q24 locus, known as rs6983267, was observed to map to a distal *cis*-regulatory element, considered to act as a transcriptional enhancer for the *MYC* proto-oncogene.^35^ The variant is associated with enhancer activation through differential binding of the beta-catenin coactivator and Wnt signaling effector TCF7L2.^35^ Furthermore, a risk SNP was identified as one of the strongest predisposing variants to neuroblastoma. The risk SNP is located within a superenhancer element, regulating the expression of LIM domain only 1 (LMO1) through the binding of the TF GATA3, which in turn facilitates long-distance enhancer–promoter looping and leads to the upregulation of LMO1.^36^

A major noncoding risk allele for prostate cancer (PCa) susceptibility and aggressiveness, rs11672691, was found to reside within an enhancer element, located in intron 2 of the lncRNA *PCAT19* locus, regulating the expression of the PCa-associated genes *PCAT19* and *CEACAM21*.^37,38^ The variant is characterized by stronger binding affinity for the TF HOXA2, which leads to the transcriptional activation of the aforementioned genes, resulting in increased proliferation and metastasis.^37^

A genetic variant located in the intron of the lncRNA *HOTAIR* (rs920778) has been observed to contribute to increased susceptibility for the development of esophageal squamous cell carcinoma and gastric cancer through a putative, de novo intronic enhancer element, leading to *HOTAIR* upregulation and further target gene expression dysregulation.^39,40^

Another intriguing example of functional enhancer variation has been reported for the obesity-associated gene *FTO* (fat mass and obesity-associated protein). SNP variants located in a 47 kb region, spanning introns 1 and 2 of the *FTO* gene, have been strongly associated with adult and childhood obesity as well as an increased risk for type 2 diabetes.^41,42^ Although SNPs in *FTO* were determined to have no effect on its expression, the 47 kb region has been demonstrated to contain multiple TF binding sites, enhancer-associated chromatin modifications, and DNase hypersensitivity sites, suggestive of the putative enhancer potential for this region. Additionally, using chromatin conformation capture technologies, the region has been observed to establish long-range interactions with the obesity-related gene *IRX3* and *FTO* risk alleles correlating with increased IRX3 expression and body mass index in humans, mice, and zebrafish.^43^

Aberrant enhancer adoption could result from genetic variations affecting TADs or TAD boundaries. An example of this can be seen in the case of autosomal dominant adult-onset demyelinating leukodystrophy (ADLD). Although ADLD has been known to be the result of genomic duplication of the lamin B (*LMNB*) gene, chromosomal rearrangements affecting TAD boundaries have been demonstrated to lead to enhancer adoption and eventual *LMNB* overexpression.^44^ Furthermore, TAD boundary disruptions and mutations in CTCF binding sites have been implicated in enhanced oncogene expression, through the formation of abnormal enhancer–promoter interactions.^45–47^

### Variation in lncRNAs

**Figure 3.**
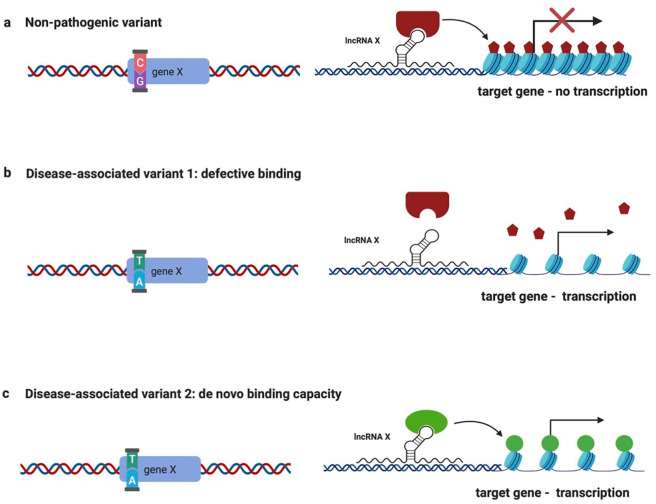
Effects of genetic variations on lncRNA functions. Graphical representation of the effects of genetic sequence variants on the function of lncRNAs. (a) The nonpathogenic variant (C allele) of gene X results in the transcription of lncRNA X, which acts as a guide for chromatin-modifying complexes (red) and represses the transcription of its target genes, by mediating repressive chromatin modifications (shown as red pentagons on histones). (b) The disease-associated variant 1 (T allele) results in the 3D changes in the structure of lncRNA X, which is unable to bind the respective chromatin-modifying protein (red), leading to de-repression and transcription of the target genes. (c) In the case of the disease-associated variant 2 (T allele), the resulting lncRNA X harbors a de novo binding site for a chromatin-modifying protein (green), leading to the recruitment of the chromatin modifier to the target genes. In this example, the chromatin modifier mediates the deposition of transcription-permissive chromatin modifications, resulting in the transcription of the otherwise repressed target genes.

During recent years, genetic variation in loci encoding for lncRNAs has been associated with the predisposition to several complex disorders. A recent study has shown that variation in genes encoding lncRNAs is more likely to include polymorphisms associated with common disorders compared with protein-coding genes.^48^ We envision three possible functional consequences regarding genetic variation in an lncRNA locus (**Fig. 3**). In physiological conditions, a locus X results in the production of the lncRNA X, which mediates the recruitment of histone modifier proteins to the target genes, leading to the deposition of repressive chromatin marks and transcriptional inhibition (**Fig. 3a**). However, in the case of genetic variation, the locus can result in the production of an lncRNA X that is unable to bind the respective chromatin modifier, leading to absence of repressive histone modification deposition and aberrant transcription of the target genes, represented in **Figure 3b**. Alternatively, we consider the possibility of the formation of a de novo binding site for a histone modifier, leading to the deposition of activating histone modifications and eventual transcriptional activation of the target gene (**Fig. 3c**).

One of the most well-known disease-associated lncRNAs is *ANRIL* (antisense noncoding RNA in the *INK4* locus). *ANRIL*, also known as the *CDKN2B-AS1* transcript, is one of at least five genes included in the 9p21 region. This chromosomal region has been identified as the strongest susceptibility region for cardiovascular disease and a hotspot for various disease-associated mutations.^49^ *ANRIL* expression and its splicing isoforms have been associated with several diseases, such as atherosclerosis, calcific aortic stenosis, myocardial infarction, type 2 diabetes, AD, glaucoma, and endometriosis.^49–54^ *ANRIL* is known to act primarily in *cis*, mediating the epigenetic silencing of *CDKN2A* and *CDKN2B* loci. Specifically, *ANRIL* has been shown to interact with PRC1 and PRC2, leading to the deposition of H3K27me3 marks on target genes.^55,56^ Because of its roles in regulating expression from the *P16/INK4* locus, *ANRIL* is considered a major regulator of mechanisms involving cell growth, proliferation, and senescence.^55,57,58^ Also, some reports demonstrate the ability of *ANRIL* to act in *trans*, regulating the transcription of genes on different chromosomes, potentially due to the presence of Alu motifs in *ANRIL* lncRNA, essential for transcriptional regulation in *trans*.^59,60^

In addition to *ANRIL*, an SNP affecting the lncRNA *MIAT* (myocardial infarction associated transcript) was found to be significantly associated with the development of myocardial infarction.^61,62^ However, unlike other lncRNAs, *MIAT* does not appear to be associated with chromatin, but is considered to be a competitive endogenous RNA (ceRNA), often termed “sponge” lncRNAs. *MIAT* has been shown to participate in negative feedback loops of the VEGF and TGFb1 pathways, by inhibiting microRNAs miR-150-5p and miR-24, respectively, resulting in the regulation of endothelial cell proliferation, migration, and apoptosis.^61–63^

*LNC13* is a recently characterized lncRNA that is involved in the inhibition of pro-inflammatory gene expression in macrophages and associated with susceptibility to celiac disease (CeD).^64^ The *LNC13* locus is located on chromosome 2 in proximity to the pro-inflammatory gene *IL18RAP* and its RNA forms a complex with HNRNPD, a ubiquitously expressed heterogeneous nuclear ribonucleoprotein and the histone deacetylase enzyme HDAC1. Upon pro-inflammatory stimulation, *LNC13* is degraded, allowing the expression of *IL18RAP*. However, in biopsies of intestinal tissue from CeD patients, *LNC13* expression is significantly reduced and is associated with the rs917997 variant characterized by inefficient binding of hnRNPD, leading to impaired transcriptional repression of *IL18RAP* and continuous pro-inflammatory gene expression.^65^

A novel lncRNA, termed *LINC00513*, was recently associated with increased susceptibility to SLE.^66^ Two variants located in the promoter region of the lncRNA, rs205764 and rs547311, were demonstrated to upregulate its expression and increase STAT1 and STAT2 phosphorylation with further activation of the IFN pathway. As its expression is significantly upregulated in patients where the disease is very active, *LINC00513* is proposed to play a major role in SLE progression and pathogenesis.^66^

The lncRNA *HOTAIR* has been extensively investigated in cancer pathogenesis and progression. Gupta and colleagues reported that upregulation of *HOTAIR* in breast cancer patients is associated with higher mortality risk through PRC2-dependent silencing of proliferation and metastasis inhibitors.^67^ Additional studies have revealed its implication in various types of cancers, such as glioblastoma,^68^ castration-resistant prostate cancer,^69^ gastrointestinal tumors,^70^ and pancreatic cancer.^71^

## A Framework for Future Studies

The emerging evidence for the role of noncoding variation in the predisposition to common diseases has highlighted the need for a new approach that combines the concepts discussed above with new technological advances.

Recent advances in genome-wide technologies to study DNA-DNA and RNA-DNA interactions in intact eukaryotic nuclei have allowed researchers to assess chromatin topology and the role of transcription in maintenance of the genome architecture. Chromatin conformation capture methodology and its later adaptations have allowed the mapping of DNA regions that are observed to be in proximity more frequently than expected, leading to the identification of the functional interaction between regulatory elements and promoters. In the past, methodologies to dissect RNA–chromatin interactions mediated by specific transcripts have identified the target genes for several lncRNAs.^72^ More recently, several technologies aimed at mapping genome-wide RNA–chromatin interactions have been developed, holding the great potential to identify the target genes of multiple transcripts at once.^73–76^ LncRNAs function through interactions with other transcripts or proteins in order to perform their biological functions. During the last decade, the advent of technologies such as RIP^77^ and CLIP^78^ has enabled the identification of multiple transcripts associated with different classes of proteins. More recently, the development of sequencing-based technologies to investigate RNA-RNA interactions at the genome-wide level has unveiled an unexpected complexity for RNA-based regulation.^79^

**Figure 4.**
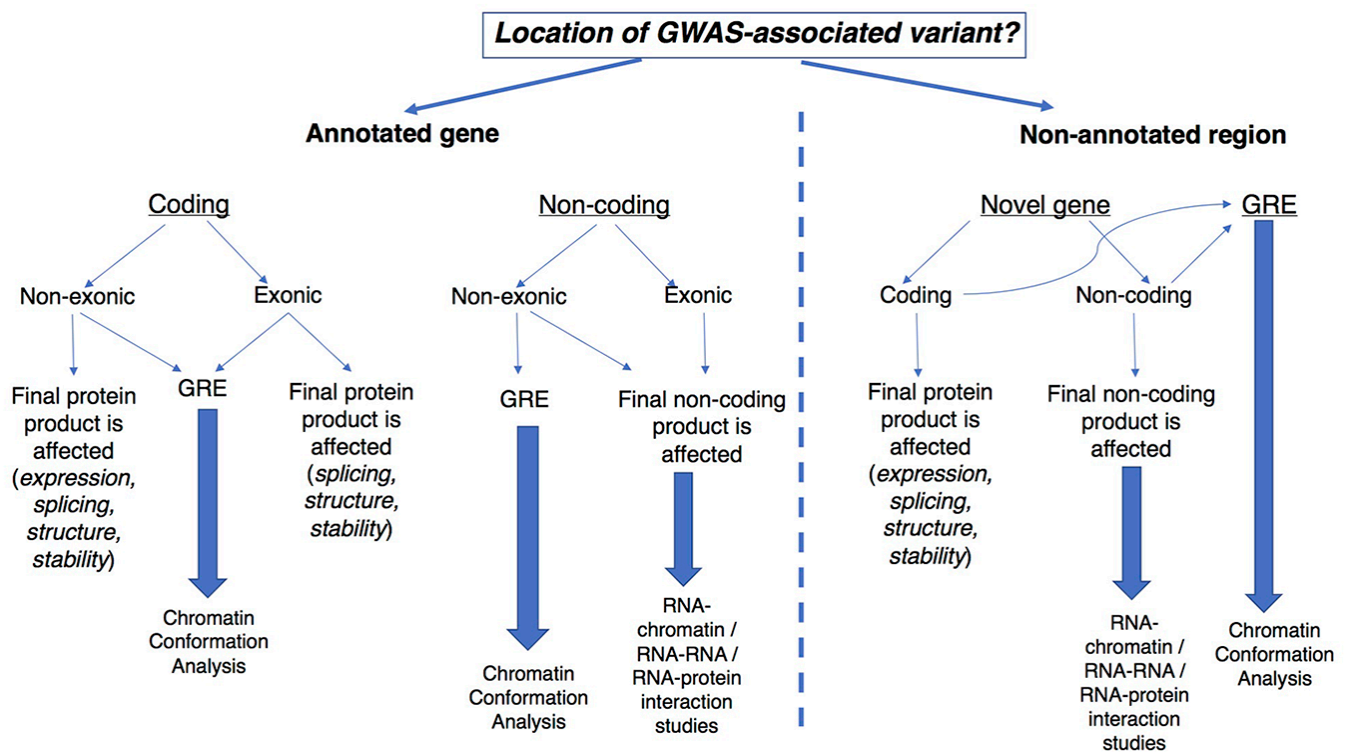
A new framework for the functional characterization of GWAS-associated noncoding variants. Schematic representation of the flowchart for the characterization of noncoding polymorphisms associated with common disorders. Details are provided in the main text.

The establishment of novel technological approaches to probe the involvement of noncoding elements at both the DNA and RNA level is quickly becoming a key feature for the identification of novel regulatory mechanisms for the control of gene expression and, consequently, cell identity (**Table 2**). We would like to propose a new framework for the functional characterization of noncoding variants associated with common diseases (**Fig. 4**). The first important step is to assess whether the variant of interest is located within an already annotated locus. Below we discuss the functional consequence of genetic variants present in different genomic regions.

**Table 2.** Technologies Employed to Functionally Characterize Noncoding Variants Associated with Common Disorders.

Technology	Target	Information
Chromatin conformation capture	Region-specific or genome-wide DNA-DNA interactions	Identification of gene targets for specific GREs
ChIRP, CHART, RAP	Transcript-specific RNA-DNA interactions	Identification of genomic targets for specific transcripts
MARGI, GRID-seq, ChAR-seq, RADICL-seq	Genome-wide RNA-DNA interactions	Identification of multiple regulatory RNAs and their genomic targets
RIP, CLIP	RNA–protein interactions	Identification of specific interactions between RNAs and proteins of interest
SPLASH, PARIS	Genome-wide RNA-RNA interactions	Elucidation of RNA structures and identification of multiple interactions among different transcripts

For each recent technological advance, the target interaction and type of retrieved information are included.

### Functional Outcome for Polymorphisms Located in Protein-Coding Loci

Polymorphisms located in protein-coding genes can have different functional outcomes depending on the gene region they are embedded in. Variants in nonexonic regions (promoter, introns, and untranslated regions) can directly affect expression levels or the stability of the transcript through various mechanisms. Furthermore, polymorphisms located in a GRE (e.g., enhancer) encompassing a nonexonic region could result in disruption of the TF binding site and, in turn, affect the expression level of nearby genes. As illustrated in **Figure 2b**, loss of TF binding can, in turn, lead to changes in 3D chromatin conformation, affecting the transcription of nearby genes. Chromosome conformation studies can identify possible target genes by pinpointing DNA interactions mediated by the region harboring the polymorphism. Examples of such an approach have led to the identification of the mechanism of action for the risk variant associated with obesity.^43^ Another possible functional outcome for noncoding variation in a GRE is disruption of the binding site for lncRNAs (from RNA–chromatin interactions technologies). Exonic variants can alter 3D contacts if encompassing a GRE. However, such variants can have a more pronounced effect by affecting either the aminoacidic sequence or the stability of the final protein product.

### Functional Outcome for Polymorphisms Located in Noncoding Loci

Polymorphisms located in noncoding genes can have different functional outcomes whether the variant acts on the DNA or RNA level. The GWAS-associated SNP can encompass a GRE and affect the binding of key TFs, disrupting possible interactions with promoter regions with functional consequences on the expression of target genes. Alternatively, the mutation can affect the sequence of the mature ncRNA, leading to structural alterations of the transcript. Technologies to identify RNA-DNA, RNA-RNA, and RNA–protein interaction can unveil novel targets for the physiological role of the RNA and its alterations in disease. Indeed, a polymorphism located in lnc13 and associated with CeD was shown to affect the interaction of the lncRNA and HNRPD protein by using RIP technology.^65^

### Functional Outcome for Polymorphisms Located in Nonannotated Regions

Functional characterization of GWAS-associated variants that map in nonannotated regions presents further challenges. As the genomic region of interest has not yet been characterized, all possible scenarios should be taken into consideration. The polymorphism could be located in a novel (coding or noncoding) gene that in turn is responsible for the observed phenotype. In this case, it is important to look into expression datasets to assess if the region harboring the variant is transcribed. Another possibility is that the variant lies within a GRE whose disruption is associated with the disorder. Indeed, characterization of a putative enhancer element revealed enhancer adoption for LMNB that in turn caused ADLD due to overexpression of the gene.^44^

### Novel Therapeutic Options for GWAS-Associated Noncoding Variants

Functional characterization of GWAS-associated noncoding variants opens new possibilities for therapeutic treatments, including the possibility for a new generation of pharmaceutical targets. Again, we need to distinguish between variants acting at the level of DNA from polymorphisms acting at the RNA level.

### Genetic Variants Acting at the DNA Level

For genetic polymorphisms associated with GWAS and functioning as GREs, it is possible to focus on the interacting gene as revealed by chromatin conformation studies. An important distinction would be to identify whether the polymorphism leads to a loss or an increase of enhancer function. This would in turn determine the nature of the pharmacological agents and their mechanisms of action (i.e., inhibitory or activating in case of hyperactivating or hypoactivating mutations, respectively). Another possibility to correct genetic variants active at DNA level is the use of new genome-editing technologies.^80-81^ One limitation for this approach is to have a delivery method that is at the same time cell type specific and does not elicit a strong immunological response.

### Genetic Variants Acting at the RNA Level

For GWAS-associated noncoding variants active at the RNA level, it is feasible to target the lncRNA by modulating its expression levels. Aberrant expression of the transcript can be reduced using gene silencing technologies such as antisense oligos (ASOs), short interfering RNAs (siRNAs), or the interference system of clustered regularly interspaced short palindromic repeats (CRISPRi).^82^ Although a delivery method to ensure cell specificity is still a limitation, the great potential of this approach has been shown in mice studies targeting the expression lncRNAs involved in human diseases.^82^ For pathological genetic variants that affect the lncRNA structure, pharmacological molecules can be envisioned to repair the 3D structure of lncRNAs. Aptamers represent a promising class of single-stranded nucleic acids that have been shown to efficiently recognize the secondary structure of specific lncRNAs and, in turn, to prevent interactions mediated by the transcript.^83^ Another possibility is to use small molecules to alter the lncRNA 3D structure and potential for interactions with DNA, other RNAs, or proteins. Albeit still in its infancy, this approach has yielded some encouraging results.^84^ Finally, the continuously expanding palette of genome-editing technologies allows researchers to edit the sequence of interest either directly in the genome or at the RNA level, with a recent approach using the CRISPR-Cas13 complex.^85^

## Conclusion

Most common disorders develop during adult life as opposed to single monogenic disorders that usually manifest at an earlier age. As most GWAS-associated genetic variants reside in regions of the genome that do not encode for proteins, the late onset of common diseases highlights the subtle regulatory role played by genetic noncoding elements.

Both enhancers and lncRNAs have restricted spatiotemporal activity patterns, making them ideal regulatory elements for the control of cell fate and coordinated gene expression during development. Recent studies have started to reveal diverse functional consequences for genetic variation located within enhancers and lncRNAs, and the availability of greatly improved or novel technologies will further expand our current knowledge on the mechanisms by which noncoding elements play a key role in human physiology and pathology.

Although still in its infancy, the development of novel pharmacological therapies targeted at noncoding elements holds great promise for the cure and treatment of common human disorders.
